# Construction of TiO_2_-Eggshell for Efficient Degradation of Tetracycline Hydrochloride: Sunlight Induced In-Situ Formation of Carbonate Radical

**DOI:** 10.3390/ma14071598

**Published:** 2021-03-25

**Authors:** Zhuquan Huang, Jiaqi Wang, Min-Quan Yang, Qingrong Qian, Xin-Ping Liu, Liren Xiao, Hun Xue

**Affiliations:** 1 College of Environmental Science and Engineering, Fujian Normal University, Fuzhou 350007, China; ZhuquanHuang0530@163.com (Z.H.); jiaqiwang77@126.com (J.W.); yangmq@fjnu.edu.cn (M.-Q.Y.); qrqian@fjnu.edu.cn (Q.Q.); lxpwkm@fjnu.edu.cn (X.-P.L.); 2 Fujian Key Laboratory of Pollution Control & Resource Reuse, Fujian Normal University, Fuzhou 350007, China

**Keywords:** photocatalysis, TiO_2_-eggshell, carbonate radical, tetracycline hydrochloride, selective oxidation

## Abstract

Photocatalytic degradation of an antibiotic by utilizing inexhaustible solar energy represents an ideal solution for tackling global environment issues. The target generation of active oxidative species is highly desirable for the photocatalytic pollutants degradation. Herein, aiming at the molecular structure of tetracycline hydrochloride (TC), we construct sunlight-activated high-efficient catalysts of TiO_2_-eggshell (TE). The composite ingeniously utilizes the photoactive function of TiO_2_ and the composition of eggshell, which can produce oxidative ·CO_3_^−^ species that are especially active for the degradation of aromatic compounds containing phenol or aniline structures. Through the synergistic oxidation of the··CO_3_^−^ with the traditional holes (h^+^), superoxide radicals (·O^2−^) and hydroxyl radicals (·OH) involved in the photocatalytic process, the optimal TE photocatalyst degrades 92.0% TC in 30 min under solar light, which is higher than TiO_2_ and eggshell. The photocatalytic degradation pathway of TC over TE has been proposed. The response surface methodology is processed by varying four independent parameters (TC concentration, pH, catalyst dosage and reaction time) on a Box–Behnken design (BBD) to optimize the experimental conditions. It is anticipated that the present work can facilitate the development of novel photocatalysts for selective oxidation based on ·CO_3_^−^.

## 1. Introduction

In recent years, the antibiotics residuals in the water environment increase gradually, which has become a serious problem for policy-makers, scientists and the public [1,2,3,4]. Tetracycline hydrochloride (TC) is one of the ten antimicrobials licensed for the promotion of livestock growth, which is now detected in significant amounts in potable, sewage water and sediment [5]. Moreover, due to its biorefractory and stable nature, the increased presence of TC in aquatic environments has raised great concerns for both environment and human health [6,7]. Therefore, developing efficient technology to eliminate TC from an aqueous environment is urgently required.

Photocatalysis is a promising strategy for environmental remediation, because it can utilize solar light directly, react at ambient temperature and has no secondary pollution [8]. The application of photocatalysis technology in the degradation of TC has been carried out. It has been found that photocatalysts such as TiO_2_, WO_3_ and C_3_N_4_ have the performance of photocatalytic degradation of TC [9,10,11,12]. At present, however, the active oxygen species involved in these photocatalytic systems are mainly hydroxyl radicals (·OH) and superoxide radicals (·O_2_^−^), which are impossible to specifically degrade TC according to its molecular structure, resulting in insufficient degradation efficiency, incomplete degradation and easy inactivation of photocatalysts [13,14,15]. Therefore, the key point towards the application of photocatalysis in the degradation of TC is, targeting to molecular structure of TC and utilizing a new degradation mechanism, to develop highly efficient sunlight responsive photocatalysts.

Carbonate radical (·CO_3_^−^) is a powerful one-electron oxidant, which displays high selectivity for the degradation of aromatic compounds containing phenol or aniline structures [16,17]. It has been observed that adding a certain concentration of CO_3_^2−^ in solution can significantly enhance the photocatalytic degradation rate of organic pollutants, such as oxytetracycline and aniline, which can be ascribed to the reaction between ·OH or holes (h^+^) produced from the photocatalysis process and HCO_3_^−^ or CO_3_^2−^ to generate ·CO_3_^−^ [18,19,20,21].

It is known to us that TC is an aromatic compound that contains the structure of phenol in detail. According to the characteristics of selective oxidation of ·CO_3_^−^, the introduction of ·CO_3_^−^ in the photocatalytic system is expected to enhance the degradation efficiency of TC. However, adding a certain amount of CO_3_^2−^ to the solution not only increases the treatment cost, but also introduces new pollutants, which may cause side reactions in the photocatalytic process. Additionally, the formation of ·CO_3_^−^ is affected by the movement rate of CO_3_^2−^, the migration and transformation of photoinduced carries and reactive oxygen species (ROS) at the solid-liquid interface [19]. Therefore, if··CO_3_^−^ can be generated in-situ on the surface of the photocatalyst, it can overcome the loss of photogenerated carriers and ROS due to its transfer and transformation at the solid-liquid interface and then improve the generation rate and yield of ·CO_3_^−^, which can greatly improve the selective oxidation ability of the photocatalyst.

TiO_2_ has been the most widely used photocatalyst due to its non-toxic, chemically stable and appropriate UV-light response range [22]. Based on the characteristics of surface hydroxylation of CaCO_3_ in the aqueous system, TiO_2_-CaCO_3_ can be constructed through the surface hydroxyl group at the phase interface of CaCO_3_ and TiO_2_. Then ·CO_3_^−^ is expected to generate in-situ on the catalyst surface [23]. Recently we reported TiO_2_–seashell composites (seashell contain more than 95% CaCO_3_) exhibited excellent solar light-driven photochemical activity in the decomposition of tetracycline hydrochloride, due to the role of ·CO_3_^−^ in promoting the degradation process [24].

As a significant byproduct of poultry eggs, eggshells contain 94–97 wt.% CaCO_3_, which are eco-friendliness, low cost, nontoxicity and easy to collect [25]. It can be used as a carrier for nanomaterials due to the randomized porous structure [26]. Preparing TiO_2_-eggshells composites cannot only generate ·CO_3_^−^ species for TC elimination under light illumination but also obtain the photocatalysts with a porous structure.

In the present work, the TiO_2_-eggshell composite was synthesized by a sol-gel method and used as a photocatalyst for the degradation of TC under solar light irradiation. The possible degradation pathway is proposed and the photocatalytic mechanism of TC degradation over TiO_2_-eggshell is validated. Response surface methodology (RSM) is applied to optimize the reaction conditions and further evaluate the interaction among the selected process parameters. This work is expected to provide a novel and practical reference for the design and preparation of selective oxidation photocatalytic materials based on in situ generation of ·CO_3_^−^.

## 2. Methods and Materials

### 2.1. Chemical and Reagents

Titanium tetraisopropoxide (C_12_H_28_O_4_Ti, 97%) was purchased from Sigma-Aldrich (Shanghai, China). Tetracycline hydrochloride (TC, 96%), 4-chlorophenol (4-CP, 99%), ammonium oxalate (AO, 99.8%), benzoquinone (BQ, 99%) and isopropyl alcohol (IPA, ≥99.5%) were provided by Aladdin (Shanghai, China). Waste eggshells were collected from a restaurant in Fuzhou, China. All chemicals were of analytical grade and used as received without further purification.

### 2.2. Synthesis Process

Eggshells with the films removed were obtained from raw chicken eggs and washed. Briefly, the collected eggshells were washed with distilled water, oven dried overnight at 80 °C for constant mass. The eggshell was calcined at 300 °C for 5 h, then the obtained power was milled, sieved to a particle size below ca.150 μm. The received powder was remarked as eggshell.

Sol-gel method was applied to synthesize TiO_2_. Firstly, titanium tetraisopropoxide was hydrolyzed in acidic conditions and further dialyzed to pH–4. In a typical combustion method, pure TiO_2_ powder was obtained through the calcination of transparent TiO_2_ sol under 300 °C for 5 h [27]. Of TiO_2_ 3.0 g was obtained by calcining 100 mL of TiO_2_ sol. The white powder was marked as TiO_2_.

To obtain the TiO_2_-eggshell composite, 1.8 g of eggshell was dispersed in 40 mL of TiO_2_ sol and stirred for 2 h at ambient temperature. After being dehydrated in a microwave oven, the gels were calcined in a muffle furnace at 5 °C/min to a fixed temperature of 300 °C and maintained for 5 h. Subsequently, the obtained powders were labeled as TE.

### 2.3. Characterization and Analytical Methods

Surface morphology of all samples was obtained by scanning electron microscopy (SEM) recorded on a Regulus 8100 field emission (Tokyo, Japan) and high resolution transmission electron microscopy (HRTEM Tecnai G2 F20, Boerne, TX, USA) at 200 kV accelerating voltage. Crystal structures of the as-synthesized samples were identified through an X-ray diffractometer (XRD) using filtered Cu-Kα radiation and scanned in the range of 10–80° at 40 kV. F(R) were measured by a CARY-100 spectrophotometer (Agilent), in which BaSO_4_ powder was used as an internal standard to obtain the optical properties of the samples over a wavelength range of 200–800 nm. The Brunauer–Emmett–Teller (BET, Osaka, Japan) specific surface area and nitrogen adsorption-desorption isotherms were carried out on a BELSORP-mini II nitrogen adsorption-desorption apparatus. Samples were pretreated at 120 °C for 2 h in vacuum to remove moisture and other gases before the test. Fourier infrared spectroscopy (FT-IR, Waltham, MA, USA) was measured in a frequency range of 4000–400 cm^−1^ on a Thermo Scientific Nicolet spectrometer. In this part, the resultant suspension was filtered through a porous membrane (0.45 μm) and the filtrate was analyzed after freeze-drying (KBr is used to press platelets).

The degradation products of TC were detected and identified by a liquid chromatography-quadrupole time-of-flight tandem mass spectrometry (LC-QTOF–MS, Malvern, UK) system equipped with an Agilent Zorbax SB-C18 (4.6 mm × 150 mm, 5 μm). Mobile phase A was aqueous formic acid solution (0.1%, *v*/*v*), and mobile phase B was acetonitrile. The gradient solvent was at a flow rate of 1 mL/min as follows: A = 90% (0 min), 75% (3 min), 65% (6 min), 60% (9 min) and 60% (12 min). The injection volume was 8 μL and the column temperature was maintained at 25 °C. The MS was performed in a positive electrospray ion mode (ESI+) under the followed conditions: fragmentor 140 V, cone voltage 65 V and nitrogen gas flow rate 10 L/min with a temperature of 350 °C. MS was full scanned by mass and this ranged from 200 to 550 *m*/*z*.

### 2.4. Photochemical Measurements

Photochemical activities of the TiO_2_-eggshell composite were evaluated by monitoring the photodegradation of tetracycline hydrochloride (TC). A 300 W Xenon lamp (PLS-SXE300, Beijing Perfect Light Co.; Beijing, China) was employed as the light source equipped with an AM 1.5 simulated solar filter (200–1200 nm). In a typical procedure, 100 mg of the as-prepared sample was dispersed in 100 mL of 50 mg/L TC aqueous solution in a glass vessel with reflux water at ambient temperature. Before irradiation, the mixed suspension was constantly stirred for 3 h in the dark to obtain absorption-desorption equilibrium. The concentration of TC at absorption–desorption equilibrium was defined as the initial concentration *C*_0_. Subsequently, the suspension was illuminated and 4 mL solution was sampled after being centrifuged immediately to remove the powder at 5 min intervals. Then the concentration of TC in the resulting clear solution was analyzed by a Shimadzu UV–vis spectrophotometer (UV-1750, San Diego, CA, USA) at a detection wavelength of 357 nm [15]. The degradation efficiency was described as Equation (1):

E% = 1 − (*C*/*C*_0_) % (1)

### 2.5. Tetracycline Hydrochloride (TC) Degradation Using Response Surface Methodology

Response surface methodology experiments were developed to optimize the reaction conditions via the Design Expert delicate product (version, 8.0.6) based on the Box–Behnken design (BBD). The total experiment set comprised four factors, pH (A), concentration (B), dosage (C) and temperature (D), each at three levels (−1, 0, 1) (Table 1). A quadratic polynomial model was applied to the response surface in terms of the four independent variables and described as Equation (2) [28,29].

(2) $$Y=b_{0}+\overset{n}{\underset{i=1}{\sum }}b_{i}x_{i}+\overset{n}{\underset{i=1}{\sum }}b_{\mathrm{ii}}x_{i}^{2}+\overset{n}{\underset{i\neq 1}{\sum }}b_{\mathrm{ij}}x_{i}x_{j}+\varepsilon$$

where Y is the predicted TC degradation efficiency, $b_{0}$, $b_{i}$, $b_{\mathrm{ii}}$ and $b_{\mathrm{ij}}$ are equal to regression constants, which represent the model, linear, quadratic and interaction coefficient, respectively. $x_{i}$ and $x_{j}$ are the independent variables in coded values, n means the number of independent variables and ε is described as the model error. In addition, the analysis of variance (ANOVA) is applied to assess the validity of the regression model, while the fit quality of the model is judged by the correlation coefficient (R^2^) and F test confirms the statistical significance.

## 3. Results and Discussion 

**Figure 1:**
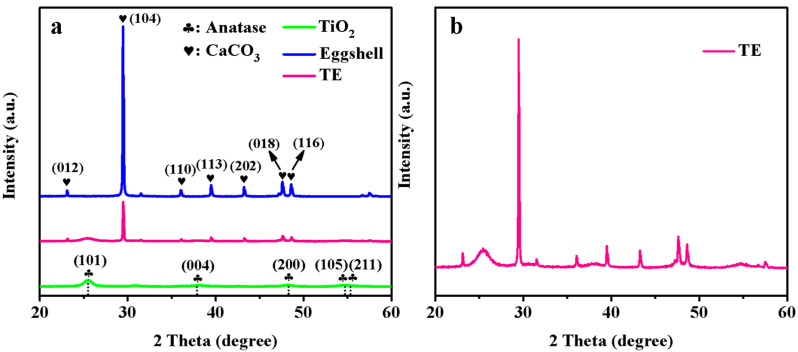
X-ray diffractometer (XRD) pattern of (**a**) the as-synthesized samples and (**b**) TiO_2_-eggshell composite (TE) in detail.

The crystal structure of TiO_2_, eggshell and TE was characterized by XRD and the results are exhibited in Figure 1. The diffraction peaks of TiO_2_ can be indexed to anatase TiO_2_ (JCPDS No. 21-1272). The correlative diffraction peaks at 25.3, 37.8, 48.1, 53.9 and 55.1° correspond to (101), (004), (200), (105) and (211) crystal planes, respectively [30]. All diffraction peaks of pure eggshell matched well with CaCO_3_ in the form of calcite, which is consistent with the previous study [31]. TE presents both the characteristic diffraction peaks of anatase TiO_2_ and CaCO_3_, demonstrating the coexistence of the two components. The result also verifies that TiO_2_ has no obvious influence on the crystal structure of eggshell. Additionally, the average crystal diameters of TiO_2_ in the samples have been calculated from the Scherrer equation based on the half-width of the (101) peak of anatase TiO_2_, which reveal that the average diameters are both around 6 nm.

**Figure 2:**
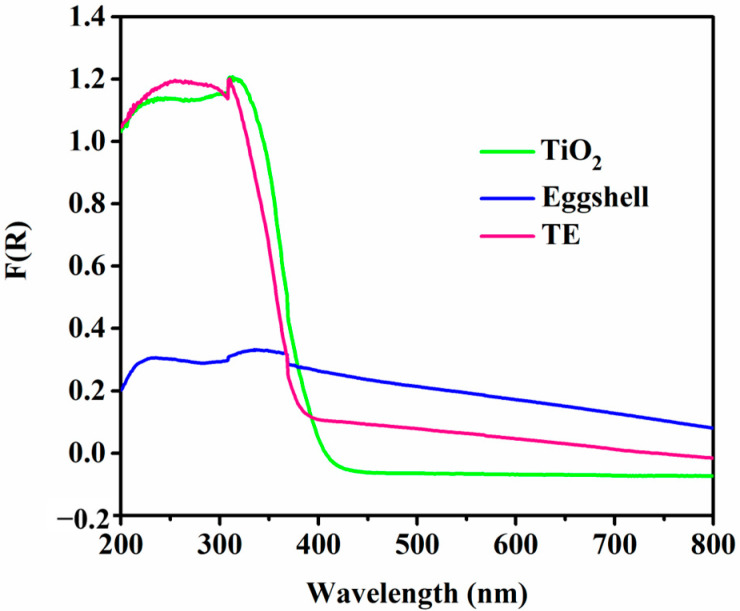
UV-visible diffuse reflectance spectra of TiO_2_, eggshell and TE.

To examine the light absorption of pure TiO_2_, eggshell and TE composite, UV-visible diffuse reflectance spectra analysis has been carried out and the result is shown in Figure 2. For pure TiO_2_, the absorption band edge was found at *ca.* 410 nm, corresponding to an intrinsic band gap of 3.02 eV. In contrast, the pure eggshell displays weak absorption in the UV-visible region without obvious absorption edge. In addition, after the hybridization of TiO_2_ with eggshell, the absorption band edge of TE is observed at 400 nm.

**Figure 3:**
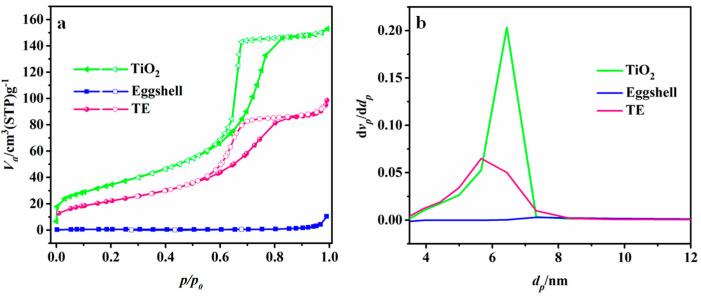
(**a**) N_2_ adsorption–desorption isotherms and (**b**) pore size distribution of the as-obtained samples.

Nitrogen physisorption measurement is applied to investigate the structure properties of TiO_2_, eggshell and TE. As shown in Figure 3, the adsorption–desorption isotherms (Figure 3a) of TiO_2_ and TE both display a type IV feature with a well-defined hysteresis loop, which is an indication of well-developed mesoporous materials. As confirmed by the Barrett-Joyner-Halenda (BJH) pore size distribution analysis, the average pore diameters (Figure 3b) were about 7.5, 34.6 and 7.5 nm for TiO_2_, eggshell and TE, respectively. As for eggshell, it shows a type II feature. The BET surface areas of TiO_2_, eggshell and TE were estimated to be 125.4, 1.8 and 91.7 m^2^·g^−1^, respectively.

**Figure 4:**
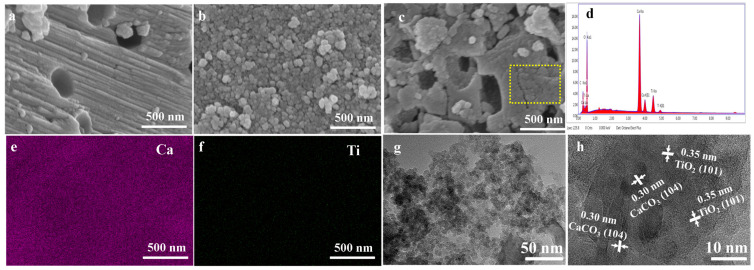
SEM images of (**a**) eggshell, (**b**) TiO_2_ and (**c**) TE; the EDS spectrum of (**d**) TE; the element mapping spectrum of a selected yellow rectangle is shown in (**c**), (**e**) Ca, (**f**) Ti; (**g**) TEM and (**h**) high resolution TEM (HRTEM) images of TE.

The SEM images of the samples are shown in Figure 4. It can be noticed that the eggshell (Figure 4a) shows a rough surface with regular stripes. Remarkably, a series of randomly distributed pores (160 nm) is observed on the surface. This is mainly caused by (i) the decomposition of organic matter; and (ii) the exposure of natural open pores of the eggshell, which has also been reported in previous study [31]. Moreover, Figure 4b of the TiO_2_ sample display a typical granulated particles morphology. The hybridization of eggshell with TiO_2_ (Figure 4c) revealed that the surface of eggshell was covered with TiO_2_ nanoparticles, establishing an intimate interfacial contact between the TiO_2_ and eggshell components. The EDS analysis of the TE (Figure 4d) shows that Ca, Ti and O elements existed in the samples, indicating that TiO_2_-eggshell compound material was successfully prepared. Additionally, the EDS element mapping analysis (Figure 4e,f) confirms the uniform distribution of Ti and Ca elements throughout the composite.

To obtain more detailed structural and micromorphology information of the TE composite, transmission electron microscopy (TEM) has been carried out. As shown in Figure 4g, the TEM image shows that TiO_2_ nanoparticles are evenly immobilized on the surface of eggshell. High resolution TEM analysis displays lattice fringes of 0.35 nm, corresponding to the (101) facet of anatase TiO_2_ [32]. Notably, the hybridization of TiO_2_ with eggshell had no obvious influence on the lattice structure of TiO_2_, as shown in Figure 4h.

**Figure 5:**
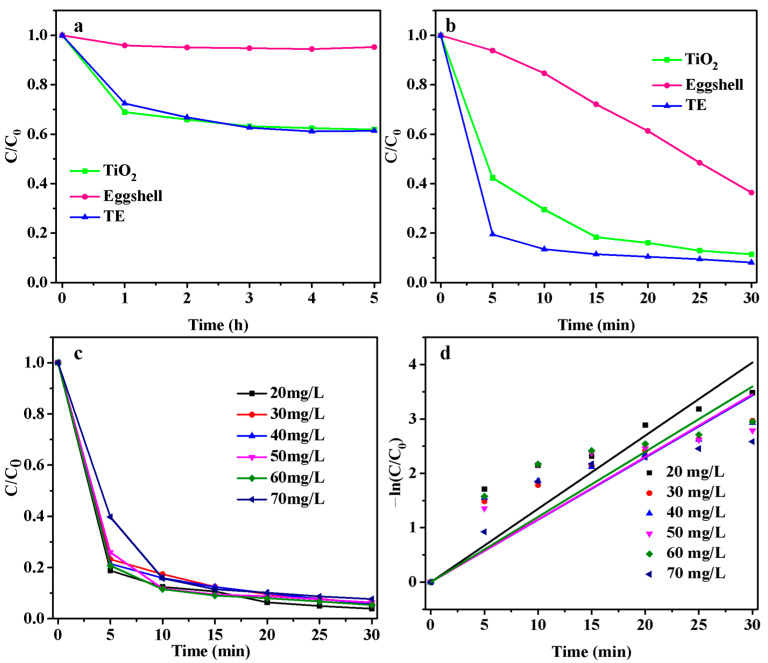
Degradation curves of tetracycline hydrochloride (TC) by the as-obtained samples (**a**) in dark conditions and (**b**) under simulated solar light illumination; the impact of initial TC concentrations (**c**) and kinetic curves of TC degradation (**d**).

**Figure 6:**
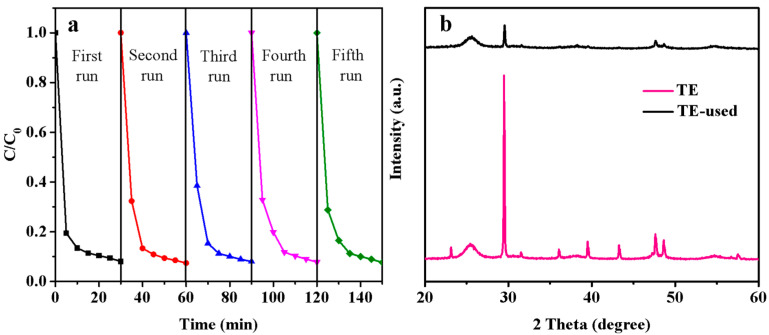
(**a**) Cycling curves of the TC over TE under solar light irradiation and (**b**) XRD pattern of TE of before and after solar light irradiation.

The photochemical activities of the as-obtained samples were evaluated by monitoring the maximal absorbance of TC at 357 nm via UV–vis spectrum under simulative solar light irradiation. Before light irradiation, the suspension was stirred for 3 h in dark to establish an adsorption-desorption equilibrium (Figure 5a). Figure 5b shows that pure TiO_2_ displays an obvious photocatalytic activity in the system with 88.6% degradation efficiency of TC. As a typical photocatalyst, the degradation ability of TiO_2_ originated from the generation of ROS, such as ·OH and h^+^ under illumination [33]. Notably, the eggshell also displays obvious photoactivity (63.7%) under simulated solar light, which is markedly more active than the case in darkness. This is mainly attributed to the fact that the abundance of CO_3_^2−^/HCO_3_^−^ in aqueous can be stimulated by the directly photolysis, which induces ·CO_3_^−^ susceptible for degradation [24]. In comparison, the TE presents the highest degradation efficiency of 92.0%, which is higher than that of TiO_2_ and eggshell. Especially in the first ten minutes of the reaction, TE showed much higher photocatalytic activity than TiO_2_ and eggshell. The significantly improved efficiency can be ascribed to the fact that the carbonate radical (·CO_3_^−^) is a transient species with high selectivity, and it has a strong redox capacity for aromatic compounds containing phenol. In addition, the intimate hybridization of TiO_2_ and eggshell helps to accelerate the formation of ·CO_3_^−^ [34,35]. Furthermore, the degradation efficiency of TC over TE drops slightly from 92.0% to 91.8% after five cycling tests, as shown in Figure 6a, which demonstrates that the TiO_2_-eggshell composite has good stability of photocatalytic activity. XRD pattern of TE of before and after solar light irradiation (Figure 6b) shows that the intensities of characteristic XRD peaks of TiO_2_ were unchanged and that of the eggshell became lower, indicating that CaCO_3_ was consumed to produced ·CO_3_^−^.

The kinetic data for the degradation of TC in the presence of eggshell, TiO_2_ and TE fit well with the pseudo-first-order model, as shown in Equation (3) and Table 2 [36].

(3) $$\ln\left(\frac{C_{0}}{C_{t}}\right)=kt$$

where $C_{0}$ is the initial concentration of TC before irradiation, $C_{t}$ is the concentration of TC at a certain interval of time (*t*) under solar light irradiation and *k* is the apparent rate constant. As shown in Table 2, the k value of the TE composite (0.226 min^−1^) was almost 10 times higher than that of eggshell (0.0216 min^−1^) and double that of TiO_2_ (0.107 min^−1^). In addition, the impact of initial TC concentration toward the photocatalytic elimination efficiency over TE was also discussed. As shown in Figure 5c, the degradation efficiency declined mildly from 96.1% to 91.3% when the initial TC concentration increased from 20 to 70 mg/L, respectively. Besides, Figure 4 exhibited that the reaction also follows a pseudo-first order kinetic model, and the k values were similar ranged from 0.1057 to 0.1347 min^−1^. Therefore, it can be deduced that the initial TC concentration had negligible influence on the photocatalytic reaction.

**Figure 7:**
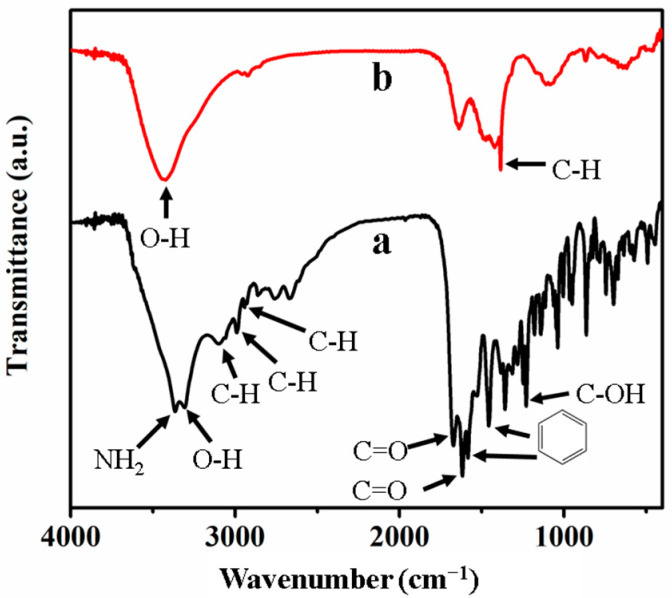
FT-IR spectra of TC (**a**) before and (**b**) after 30 min photodegradation.

The intermediates generated during the TC degradation was measured by FT-IR spectra. As shown in Figure 7, before the photodegradation with TE, the broad peaks centered at 3364 and 3307 cm^−1^ were attributed to NH_2_ symmetric stretching vibrations and O–H stretching vibrations on TC. The absorption peak at 3099 cm^−1^ was associated with C–H stretching vibration of aromatic hydrocarbon. Together, the peaks at 2963 and 2930 cm^−1^ were respectively assigned to C–H(CH_3_) and C–H(CH_2_) antisymmetric stretching vibrations. The peaks at 1671 and 1228 cm^−1^ were linked to the carbonyl group and C-OH stretching vibration, respectively, while peaks at 1616 cm^−1^ could be inferred to the carbonyl group in the hexatomic ring. Furthermore, the 1582, 1456 and 1382 cm^−1^ bands constitute the skeleton vibration of the aromatic −C=C−.

In contrast, after light irradiation, the spectrum (Figure 7) of the degraded TC became smooth, indicating that the functional groups of tetracycline were decomposed or removed. The peaks at 3364 and 3307 cm^−1^ disappeared. Meanwhile, a broad peak that assigned to hydroxyl stretching vibrations in moisture appeared at 3423 cm^−1^, suggesting that the amine and hydroxyl group in phenol was eliminated. Moreover, the flexural vibrational peak of C-H at 1384 cm^−1^ was apparently weakened. Additionally, the peaks at 1616, 1582 and 1456 cm^−1^ completely disappeared after the reaction, denoting the removal of carbonyl group and aromatic −C=C−.

**Figure 8:**
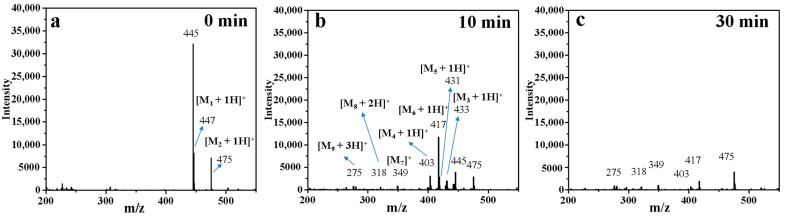
LC-QTOF–MS mass spectra of transformation by products during the photodegradation of TE at different reaction times.

**Scheme 1:**
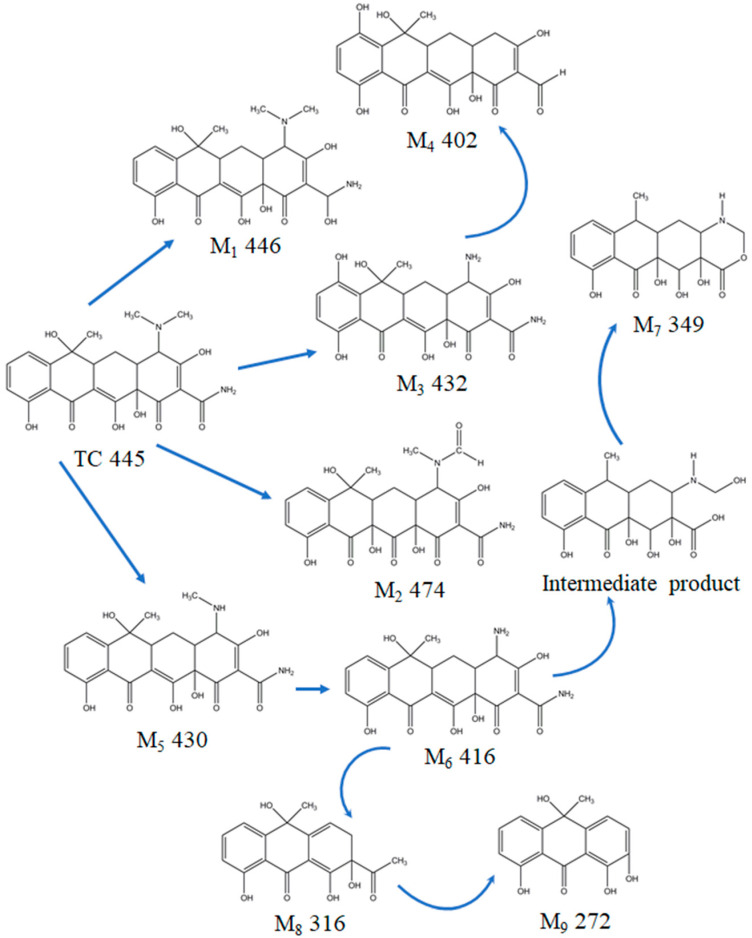
Suggested photodegradation pathway of TC.

To further investigate the intermediates generated in the system, LC-QTOF–MS was undertaken, as shown in Figure 8. It is obvious that the intensity of different products was weakened and even disappeared in 30 min. A possible degradation pathway of TC was proposed in Scheme 1. Compared to parent TC, M_1_ (*m*/*z* 447) may be generated from TC via a hydroxylation process occurring on the carbonyl, which is consistent with a recent study [37]. Besides, M_2_ (*m*/*z* 474) was generated from TC via hydroxylation and subsequently subjected to attack by reactive radicals, which has been documented in Yang et al.’s study [38]. Similarly, M_3_ (*m*/*z* 432) was identified and arose from the hydroxylation and the removal of two N-methyl groups, respectively, which further led to the generation of M_4_ (*m*/*z* 402) through detachment of two amino groups. This is due to the weak bond energy of N-C [39]. Additionally, the generation of M_5_ (*m*/*z* 430) and M_6_ (*m*/*z* 416) were attributed to successive N-dealkylation steps of TC. On one hand, after the reaction of dehydroxylation, the C–C double bond of M_6_ was attacked by the reactive species in the system, and finally, the intermediate product degrades to M_7_ (*m*/*z* 349) through intramolecular condensation, which has been described in a previous research [37]. On the other hand, the M_6_ can be further fragmented into M_8_ (*m*/*z* 316) via dehydroxylation, deamination, opening of the hexatomic ring, deethylation and additive reaction. Then the M_8_ is transformed into M_9_ (*m*/*z* 272) via deacetylation. A similar degradation pathway has been suggested in the recent research of Yang et al [38].

**Figure 9:**
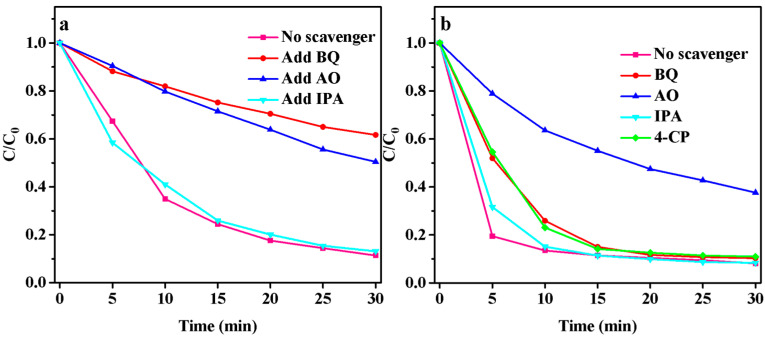
Active species trapping experiment on TC degradation in the presence of (**a**) TiO_2_ and (**b**) TE.

Identifying the main reactive radicals is important to reveal the mechanism of the TC degradation over the TE composite. Different radical scavengers of AO (a quencher of h^+^), BQ (a quencher of ·O_2_^−^), IPA (a quencher of∙·OH) and 4-CP (a quencher of∙·CO_3_^−^) have been added into TC aqueous solution, respectively [18,40]. As shown in Figure 9, the photoactivity of TiO_2_ shows a dramatic reduction from 88.6% to 38.4%, and to 49.6% after the addition of BQ and AO, respectively (Figure 9a), implying that ∙O_2_^−^ and h^+^ play dominant roles in the degradation of TC when using TiO_2_. However, fuzzy reduction is observed after adding IPA, which suggests that the ·OH radical plays a minor role in the process.

In comparison, the TE displays a different mechanism for the degradation of TC. As depicted in Figure 9b, when BQ and AO were added, the photodegradation efficiency of TC declined from 92.0% to 89.8% and 62.4%, respectively. Moreover, the degradation efficiency was also clearly worse than after adding IPA, which indicates that the ·OH was also involved in the photoreaction process, from the result it could be claimed that the consumption of ·CO_3_^−^ could promote the generation of ·OH to some extent. Furthermore, when using the TE composite directly, the 4-CP capturer inhibited the degradation activity of TC obviously, which implied that the ·CO_3_^−^ radical also plays an important role in this photocatalytic system. These results were consistent with the previous photodegradation research that involves the ·CO_3_^−^ species [18]. Therefore, the generation of ·CO_3_^−^ radical and the synergistic effect of ·CO_3_^−^, h^+^, ·O_2_^−^ and ·OH contribute to the significantly improved degradation efficiency.

Results of the statistical analysis are detailed in Table 3. An empirical relationship between response and variables was found. The result was in good accordance with the experiment data, for which the TC degradation efficiencies ranged from 24.6 to 96.9%. The most appropriate method to arrive at a final model via regression analysis in this photochemical degradation system can be ascribed to Equation (4):

Y = −0.851 + 0.025 × A + 2.840 × B + 0.362 × C − 0.012 × D − 0.106 × AB + 1.04E − 03 × AC + 1.99E − 04 × AD + 0.039 × BC + 0.087 × BD + 5.03E − 04 × CD − 5.42E − 04 × A^2^ − 26.055 × B^2^ − 0.023 × C^2^ − 4.72E − 05 × D^2^ (4)

where Y is the efficiency of TC degradation, A is irradiation time, B is catalyst dosage, C is pH and D is initial concentration. Additionally, analysis of variance (ANOVA) for the quadratic polynomial regression equation of predicted values is also depicted in Table 3. All parameters in this model are ascertained via ANOVA (Table 4). The F value (9.7) and *p* values (<0.0001) suggest that the model could well explain the relationships between TC degradation efficiency and the four system parameters mentioned above. Furthermore, the F values of A (*p* < 0.0001), AB (*p* = 0.0233), BD (*p* = 0.0005), A^2^ (*p* = 0.0114), B^2^ (*p* = 0.0004) and C^2^ (*p* = 0.0014) were also statistically significant model terms. The good correlation between the experimental and standard estimations (R^2^ = 0.906) means that the selected factors (irradiation time, dosage, pH and concentration) and regression model exhibited a good prediction of TC degradation efficiency in aqueous solution. Pursuant to this model, the effect of respective factors on the photodegradation of TC by TE was proceeded as follows: dosage > irradiation time > pH > concentration. Here the optimal photodegradation condition was stated: irradiation time of 27 min, catalyst dosage of 0.15 g, pH of 8.4 and initial concentration of 78 mg/L, yielding a high degradation rate for TC of 99.9%. It can also be asserted that the interaction between catalyst dosage and irradiation time had the largest effect on the photodegradation of TC by TE.

**Figure 10:**
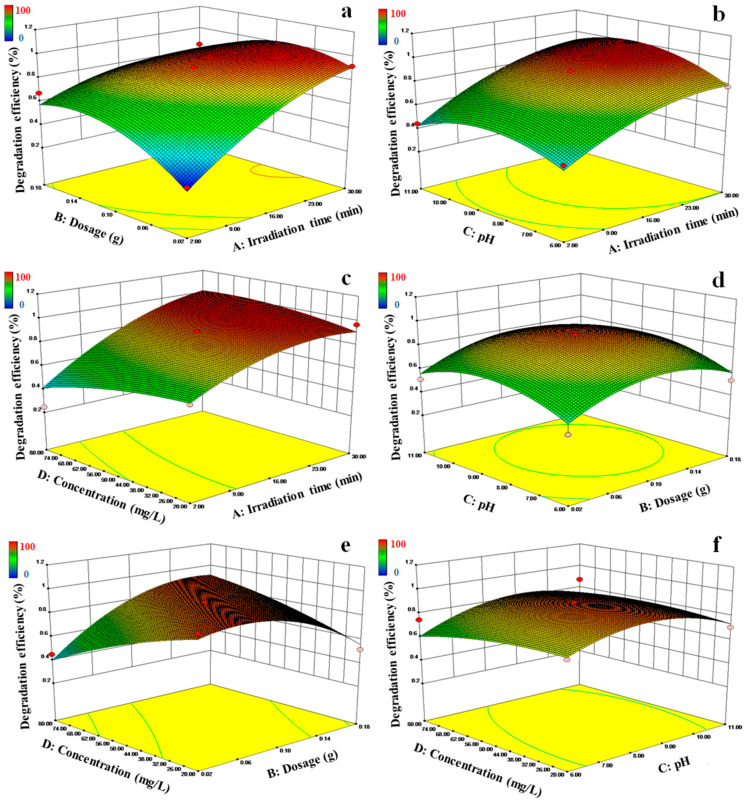
Response surfaces for TC degradation: (**a**) irradiation time–dosage, (**b**) irradiation time–pH, (**c**) irradiation time–concentration, (**d**) dosage–pH, (**e**) dosage–concentration and (**f**) pH–concentration.

The contour nature of Figure 10 shows the relationships in terms of operating parameters, which comprises irradiation time, catalyst dosage, pH, TC concentration and their effect on TC degradation efficiency at room temperature (25 °C). As shown in Figure 10a, insufficient TE dosage and low irradiation time does not encourage TC degradation. The photodegradation rate of TC increases comparably when the pH < 8.5 but fell away considerably when the pH > 8.5 regardless of irradiation time (Figure 10b). A large amount of TC is decomposed when the irradiation time increased with the indicated concentrations ranges from 20 to 80 mg/L (Figure 10c). The photodegradation rate of TC apparently improved when the pH < 8.5, yet this was inhibited considerably when the pH > 8.5, this refers to the change in catalyst dosage from 0.02 to 0.18 g (Figure 10d). It is notable that degradation efficiency increased when the catalyst dosage <0.1 g but declined significantly when the catalyst dosage >0.1 g, when the concentrations were increased (Figure 10e). Better degradation efficiency of TC was obtained at pH < 8.5 with the initial concentration ranging from 20 to 80 mg/L. However, degradation efficiency declined when the pH was above 8.5 (Figure 10f). Collectively, the results demonstrate that each two different variables had quite different effects on the degradation rate of TC, and the interaction among the selected process parameters were confirmed.

## 4. Conclusions

In this research, a novel TiO_2_-eggshell (TE) material was successfully fabricated as an efficient catalyst for the targeted degradation of tetracycline hydrochloride (TC) under solar light irradiate. A possible photodegradation pathway of TC was proposed, which suffered deamination, dehydroxylation or removal of other functional groups. The observed high efficiency of TE was mainly ascribed to the in-situ formation of carbonate radical (·CO_3_^−^) and the synergistic effect of ·CO_3_^−^, h^+^, ·O_2_^−^ and ·OH. The best degradation condition for TC was at an initial concentration of 78 mg/L, pH of 8.4, TE dosage of 0.15 g and irradiated by simulated solar light for 27 min. The corresponding degradation ratio reached 99.9%. The present work shows that the simple yet effective TE composite had great potential for targeted remediating antibiotics from wastewaters under solar light illumination.

## Figures and Tables

**Table 1:** Independent variables and levels used for response surface methodology (RSM).

Factors	Levels		
	Low (−1)	Medium (0)	High (+1)
A: Irradiation time (min)	2	16	30
B: Dosage (g∙L^−1^)	0.02	0.1	0.18
C: pH	6	8.5	11
D: Concentration (mg∙L^−1^)	20	50	80

**Table 2:** Kinetics parameters of the pseudo-first-order model for TC degradation by the as-prepared samples.

Samples	Degradation (%)	k (min^−1^)	R^2^
Eggshell	63.7	0.0216	0.993
TiO_2_	88.6	0.107	0.979
TE	92.0	0.226	0.999

**Table 3:** Response center combination test design and results.

Run	A	B	C	D	TC Degradation Efficiency (%)	
					Observed	Predicted
1	16	0.1	6	20	73.0	77.1
2	30	0.1	8.5	80	91.6	108.4
3	2	0.1	8.5	20	63.5	67.4
4	30	0.1	11	50	89.9	99.4
5	2	0.02	8.5	50	27.0	28.1
6	16	0.1	8.5	50	89.9	94.4
7	16	0.1	11	80	87.3	82.0
8	30	0.1	8.5	20	96.9	95.0
9	30	0.18	8.5	50	85.0	82.3
10	16	0.1	8.5	50	89.8	94.4
11	16	0.02	8.5	20	92.2	91.1
12	16	0.1	8.5	50	89.7	94.4
13	16	0.1	8.5	50	89.8	94.4
14	2	0.1	11	50	44.3	47.7
15	16	0.02	6	50	42.8	55.0
16	30	0.02	8.5	50	92.0	96.2
17	30	0.1	6	50	78.1	83.8
18	16	0.1	8.5	50	89.7	94.4
19	2	0.18	8.5	50	67.3	61.7
20	16	0.1	6	80	74.7	66.1
21	16	0.18	8.5	20	51.5	59.2
22	16	0.18	11	50	64.4	73.2
23	16	0.02	8.5	80	45.0	46.0
24	2	0.1	6	50	47.2	46.7
25	2	0.1	8.5	80	24.7	47.3
26	16	0.18	6	50	52.9	63.3
27	16	0.1	11	20	70.5	77.9
28	16	0.02	11	50	51.3	61.8
29	16	0.18	8.5	80	87.6	97.6

**Table 4:** The response surface quadratic model via ANOVA for the photodegradation of TC.

Source	Sum of Squares	DF	Mean Square	F Value	*p*-Value	
					Prob > F	
Model	1.17	14	0.084	9.7	<0.0001	significant
A	0.56	1	0.56	65.02	<0.0001	
B	0.029	1	0.029	3.31	0.0902	
C	0.013	1	0.013	1.47	0.2459	
D	0.011	1	0.011	1.29	0.2751	
AB	0.056	1	0.056	6.48	0.0233	
AC	5.32 × 10^−3^	1	5.32 × 10^−3^	0.62	0.4458	
AD	0.028	1	0.028	3.24	0.0936	
BC	2.42 × 10^−4^	1	2.42 × 10^−4^	0.028	0.8696	
BD	0.17	1	0.17	20.1	0.0005	
CD	5.70 × 10^−3^	1	5.70 × 10^−3^	0.66	0.4303	
A^2^	0.073	1	0.073	8.47	0.0114	
B^2^	0.18	1	0.18	20.88	0.0004	
C^2^	0.14	1	0.14	15.78	0.0014	
D^2^	0.012	1	0.012	1.36	0.2635	
Residual	0.12	14	8.64 × 10^−3^			
Lack of Fit	0.12	10	0.012			
Pure Error	0	4	0			
Cor Total	1.29	28				
R^2^			0.906			

## Data Availability

All data is contained within the article.
